# Proton-Sensing GPCRs in Health and Disease

**DOI:** 10.3390/cells10082050

**Published:** 2021-08-10

**Authors:** Marco Sisignano, Michael J. M. Fischer, Gerd Geisslinger

**Affiliations:** 1 Institute of Clinical Pharmacology, Pharmazentrum Frankfurt/ZAFES, University Hospital of Goethe-University, Theodor-Stern-Kai 7, 60590 Frankfurt am Main, Germany; geisslinger@em.uni-frankfurt.de; 2 Fraunhofer Institute for Translational Medicine and Pharmacology (ITMP), Theodor-Stern-Kai 7, 60596 Frankfurt am Main, Germany; 3 Center for Physiology and Pharmacology, Institute of Physiology, Medical University of Vienna, Schwarzspanierstrasse 17, 1090 Vienna, Austria; michael.jm.fischer@meduniwien.ac.at; 4 Fraunhofer Cluster of Excellence for Immune-Mediated Diseases (CIMD), Theodor-Stern-Kai 7, 60596 Frankfurt am Main, Germany

**Keywords:** proton-sensing GPCR, inflammation, pain, neuropathic pain, GPCR, tumor microenvironment, GPR4, TDAG8, OGR1, G2A

## Abstract

The group of proton-sensing G-protein coupled receptors (GPCRs) consists of the four receptors GPR4, TDAG8 (GPR65), OGR1 (GPR68), and G2A (GPR132). These receptors are cellular sensors of acidification, a property that has been attributed to the presence of crucial histidine residues. However, the pH detection varies considerably among the group of proton-sensing GPCRs and ranges from pH of 5.5 to 7.8. While the proton-sensing GPCRs were initially considered to detect acidic cellular environments in the context of inflammation, recent observations have expanded our knowledge about their physiological and pathophysiological functions and many additional individual and unique features have been discovered that suggest a more differentiated role of these receptors in health and disease. It is known that all four receptors contribute to different aspects of tumor biology, cardiovascular physiology, and asthma. However, apart from their overlapping functions, they seem to have individual properties, and recent publications identify potential roles of individual GPCRs in mechanosensation, intestinal inflammation, oncoimmunological interactions, hematopoiesis, as well as inflammatory and neuropathic pain. Here, we put together the knowledge about the biological functions and structural features of the four proton-sensing GPCRs and discuss the biological role of each of the four receptors individually. We explore all currently known pharmacological modulators of the four receptors and highlight potential use. Finally, we point out knowledge gaps in the biological and pharmacological context of proton-sensing GPCRs that should be addressed by future studies.

## 1. Proton-Sensing GPCRs—Structural Features and Physiology

The family of proton-sensing G-protein coupled receptors (GPCRs) has first been described by Ludwig et al. in 2003 and consists of four members that belong to the class A orphan GPCRs: GPR4, TDAG8 (GPR65), OGR1 (GPR68), and G2A (GPR132) [1,2,3]. Under physiological conditions, GPR4, OGR1, and G2A are expressed ubiquitously, although G2A shows the strongest expression in leukocytes, such as T- and B-cells, neutrophils, and macrophages [4,5,6]. In contrast, TDAG8 is expressed almost exclusively in lymphoid tissue [7,8].

**Figure 1:**
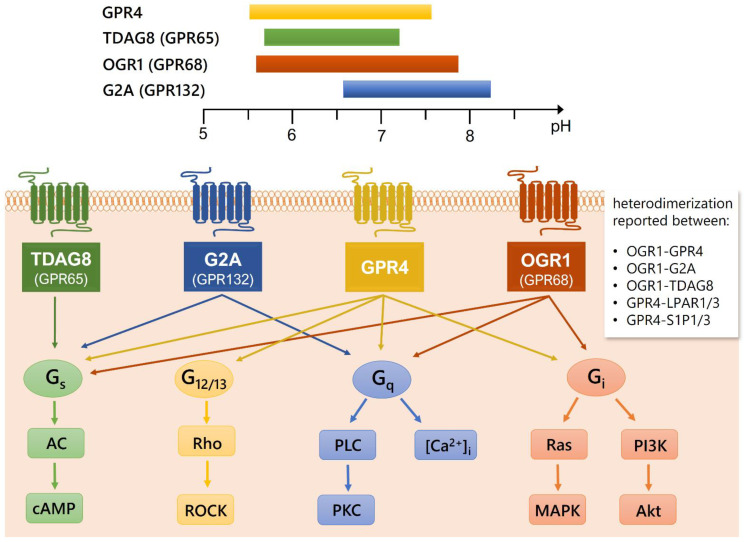
pH activation range in vitro, coupling preferences and signaling of the four proton-sensing GPCRs. The individual pH activation range for each of the four receptors TDAG8 (GPR65, green), G2A (GPR132, blue), GPR4 (yellow), and OGR1 (GPR68, red) is shown above. The pH ranges were determined in heterologous expression systems using various cell lines and are obtained from [1,5,42]. The receptors can couple to different G-proteins (G_S_, G_12/13_, G_q_, G_i_). The coupling preferences for each receptor differ depending on the investigated cell type, tissue, and the physiological or pathophysiological state, and were obtained from [1,8,9,10,11,14,43,44,45]. Abbreviations: AC: adenylyl cyclase, cAMP: cyclic adenosine monophosphate, Rho: Ras homologue, ROCK: Rho-associated coiled-coil-containing protein kinase, PLC: phospholipase C, PKC: protein kinase C, [Ca^2+^]_i_: intracellular calcium concentration, Ras: rat sarcoma protein, MAPK: mitogen-activated protein kinase(s), PI3K: phosphoinositide 3-kinase, Akt: protein kinase B, LPAR: lysophosphatidic acid receptor, S1PR: sphingosine-1-phosphate receptor.

Upon activation, the four receptors seem to activate different signaling pathways [9,10,11] depending on the investigated cell type. It has also been reported that OGR1 and GPR4 can form heterodimers with each other, with G2A and TDAG8, and with other receptors [12] (Figure 1). Heterodimerization can have a variety of functional consequences, such as increased or decreased activation by a ligand, as well as co-endocytosis, transactivation, or transinhibition [13]. However, these effects have not yet been studied for heterodimers of the proton-sensing GPCRs and the functional consequence of these heterodimerizations for cellular signaling processes remains unknown.

Despite the lack of structure at the atomic level for any of the four GPCRs, analysis of the amino acid sequence and site-directed mutagenesis revealed the presence of crucial histidine residues that seem to be responsible for the detection of increased extracellular proton concentrations and the activation of the receptors upon pH reduction [1]. More specifically, protonation of the imidazole group of these histidines causes loosening and destabilization of hydrogen bonds among the histidines and may eventually change the receptor conformation to an active state [1].

Interestingly, these histidine residues are not conserved equally among all members of the proton-sensing GPCR-family. While GPR4 and OGR1 have the strongest amino acid similarity, TDAG8 and especially G2A seem to differ in their amino acid sequence and histidine distribution [5,14]. These structural differences indicate that the individual members of the proton-sensing GPCR family respond differently to pH changes in the extracellular environment. Indeed, the G2A receptor seems to be less sensitive to pH changes than the other three members of the proton-sensing GPCR family, probably due to the lack of crucial histidines at the respective positions [8]. Moreover, a recent publication suggested a triad of acidic amino acids for proton detection that are conserved in GPR4, OGR1, and TDAG8, but not in G2A, which may additionally explain the weak proton activation of G2A, and which provides molecular evidence for the evolution of proton-sensing GPCRs allowing phylogenetic classification of the receptors [15].

Upon discovery, the physiological and pathophysiological role of proton-sensing GPCRs was unclear. It was speculated that these receptors are activated in the context of tissue injury and inflammation where local acidosis occurs and the pH in the microenvironment decreases. Indeed, proton-sensing GPCRs have been found to be involved in these processes [16,17].

However, several lines of evidence point towards the role of these receptors in the tumor microenvironment. A hallmark of tumor cells is the so-called inversed pH gradient, which means, that tumors become more acidic extracellularly and more alkaline intracellularly compared with healthy cells. This effect is mainly mediated by the aberrant activity of the Na+/H+ exchanger NHE1 and other membrane-bound acid extruding proteins in tumor cells [18]. Apart from that, cellular metabolism is disturbed causing enhanced glycolysis as well as the Warburg effect, a metabolic adaptation that leads to ATP synthesis by the non-oxidative breakdown of glucose to lactic acid [19]. In this regard, cellular sensors of the surrounding pH, such as integrins and the proton-sensing GPCRs, are important mediators of tumors and the tumor microenvironment.

The metabolic interactions of tumor cells and immune cells in their proximity are complex but mostly result in activity suppression of NK cells, macrophages, and other immune cells. For example, tumor cells deplete nutrients that are also required by adjacent immune cells and, in turn, secrete lactate, fatty acids, and reactive oxygen species, the latter of which are generated by excessive oxidative phosphorylation of tumor cells. This aberrant metabolic activity of tumor cells suppresses metabolism and normal cellular functions in neighboring immune cells. The concept of modulating the metabolism of immune cells in the tumor-microenvironment to regain their full anti-tumor activity could provide new therapeutic approaches in cancer [20,21].

Beyond the scope of the review is a detailed account of other pH-sensing mechanisms [22]. Briefly, most of these are ion channels, and the reader is referred to respective reviews [23,24]. Among these, transient receptor vanilloid 1 channel (TRPV1) is the principal receptor for a human pH 6-induced nociception [25].

What applies to all pH-sensors, is to consider their pH-working range, including the threshold, and the suprathreshold pH response-function which can saturate or decrease beyond the optimal working range.

A further important parameter is the extent of desensitization to prolonged stimulation, the latter is common in pathophysiological processes [26]. The desensitization of GPCRs is comprised of several mechanisms [27] including the rapid phosphorylation by G-protein receptor kinases and second messenger kinases and uncoupling of the receptor from the G-proteins [28]. Desensitization of a single GPCR can be agonist-dependent, which includes different downstream pathways and time courses, as e.g., outlined at the µ opioid receptor by PKC-dependent desensitization via morphine vs. GRK-dependent desensitization by DAMGO [29]. This can lead to a change in affinity [30], but also internalization, which is further facilitated by e.g., β-arrestin [31]. Arrestins are scaffolding proteins that target the receptors towards clathrin-coated pits, from where they are internalized. In cases without reduced receptor affinity (Kd constant), membrane presence can be temporarily reduced or depleted, and incomplete recycling due to lysosomal breakdown can lead to a lower-level steady state. However, for the proton-sensing GPCRs, there is little evidence for the strong involvement of β-arrestin recruitment upon receptor activation [32]. For G2A, a β-arrestin induction has been proposed which seems to depend on its activation by lysophospholipids and not by the proton-induced activation [33].

Long-term exposure to an agonist causes a homologous (partial) desensitization of a specific GPCR [34]. In addition, there is heterologous desensitization, where another pathway leads to GPCR desensitization [35]. Regarding desensitization, the picture is incomplete for the proton-sensing GPCRs.

Moreover, recent studies suggest important roles of the proton-sensing GPCRs in pathophysiological contexts other than acidification in the context of inflammation [36,37,38,39,40,41]. Hence, the biological role of proton-sensing GPCRs is much broader and more complex than initially suggested. Here, therefore, we discuss recent observations concerning physiological and pathophysiological roles with a focus on the individual receptors.

## 2. GPR4

The GPR4 receptor is widely expressed in different tissues and was initially described to be a proton-sensing GPCR by Ludwig et al. in 2003 [1]. It is activated over a broad pH range from 5.6–7.6 and shares all the crucially involved histidine residues with OGR1. A mutational study revealed that each of the histidine residues His79, 165, and 269 of GPR4 are required for proton-dependent activation, leading to elevated cAMP-concentrations via G_s_-coupling [45]. It was reported that GPR4 can be activated by lysophospholipids (LPC), however, this paper was later retracted [46]. It was later suggested that LPC may instead modulate its translocation to the plasma membrane within the cell. Indeed, the LPC-stimulated endothelial barrier dysfunction seems to be mediated through GPR4 [47].

Several roles have been suggested for GPR4 during inflammation. Generally, GPR4 seems to aggravate inflammation and leukocyte adhesion to endothelial cells via G_s_-cAMP-Exchange protein activated by cAMP (Epac) activation [48], via nuclear factor kappa-light-chain-enhancer of activated B-cells (NF-κB) and cyclooxygenase-2 (COX-2) induction [49]. Additionally, GPR4-deficiency reduces intestinal inflammation in a murine colitis model [38,50] and in a model of inflammatory bowel disease (IBD) [51]. In human vascular endothelial cells, activation of GPR4 causes endoplasmic reticulum (ER) stress via activation of the unfolded protein response (UPR) proteins PKR-like ER kinase (PERK), inositol requiring enzyme 1α/β (IRE1) and activating transcription factor 6 (ATF6) [52]. While these reports indicate a proinflammatory role of GPR4, its deficiency was also associated with reduced renal acid excretion and caused metabolic acidosis in the kidney, which implies a central role of GPR4 in physiological renal acid clearance [53].

In inflammatory pain, peripheral sensory neurons are sensitized by several mediators that are released from the resident or invading immune cells. Among these sensitizing mediators are protons, causing local acidification on the surface of primary afferent sensory neurons [54]. GPR4 is expressed in sensory neurons in dorsal root ganglia (DRGs) expressing the transient receptor potential vanilloid 1 channel (TRPV1) [55], and its expression in DRGs increased significantly 24 h after injection of carrageenan [56].

The role of GPR4 in angiogenesis and tumor biology seems to be more contradictory. On the one hand, GPR4 seems to be pro-oncogenic and overexpressed in human tumors [57,58,59,60]. Moreover, it was described to promote pathological angiogenesis and tumor growth via p38-mediated interleukin- (IL-) 6, IL-8, and vascular endothelial growth factor-a (VEGF-A) secretion after pH decrease [61,62]. However, GPR4 can also reduce angiogenesis and seems to be required for normal vessel formation under physiological conditions [63]. Such a requirement for an essential function might well allow for a short-term manipulation as a pharmacological concept but has to inflict side effects in a prolonged or continuous manner. Likewise, GPR4 can reduce migration and metastasis of mouse B16F10 melanoma- and TRAMP-C1 prostate cells in vitro [64] but does not inhibit B16F10 melanoma growth [65]. It seems that, depending on the tumor microenvironment and cellular context, GPR4 can be involved in both tumor-promoting and tumor-suppressing processes.

**Figure 2:**
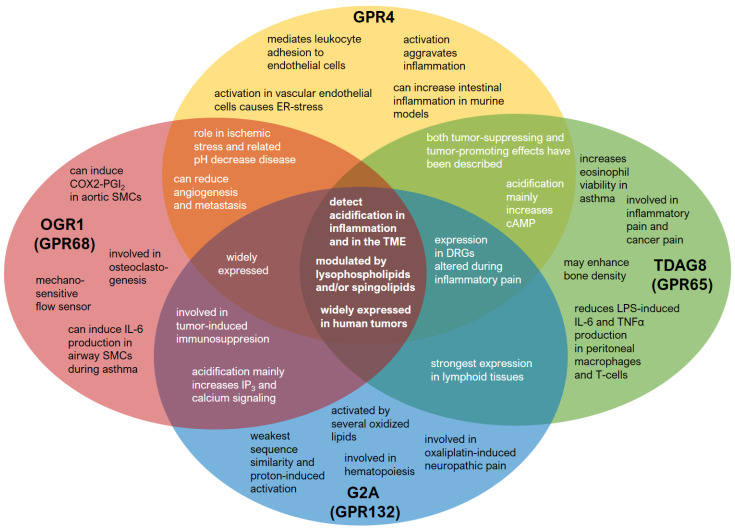
Individual and overlapping physiological and pathophysiological properties of the four proton-sensing GPCRs. GPR4 (yellow), TDAG8 (GPR65, green), G2A (GPR132, blue), and OGR1 (GPR68, red). The figure visualizes functional overlaps between the proton-sensitive GPCRs. The actual pairwise overlap between these receptors in sensory neurons has been demonstrated previously [55]. Abbreviations: ER: endoplasmic reticulum, cAMP: cyclic adenosine monophosphate, DRG: dorsal root ganglia, LPS: lipopolysaccharide, IL-6: interleukin 6, TNFα: tumor necrosis factor α, TME: tumor microenvironment, SMC: smooth muscle cells, COX-2: cyclooxygenase-2, PGI_2_: prostaglandin I_2_.

In the cardiovascular context, GPR4 was shown to be activated upon ischemic stress and pH decrease, and GPR4 may influence myocardial infarction originated from ischemic stress and subsequent pH decrease (Figure 2). In this context, a dibenzazepine derivate has been described as a potent GPR4 antagonist that reduced mortality in a mouse myocardial infarction model in which mice were subjected to permanent ligation of the left anterior descending coronary [66].

GPR4 is also expressed in the brain, specifically in the cerebrovascular endothelium dorsal raphe neurons, and neurons in the retro-trapezoidal nucleus, locus coeruleus, and the lateral septum [67]. Recently, a connection between GPR4 and cerebrovascular reactivity was found. In GPR4 deficient mice, the CO_2_-induced blood flow stimulation was reduced, indicating a role for endothelial GPR4 via G_αq/11_ signaling in CO_2_-induced vascular response [68].

Until now, several orally active GPR4 inhibitors have been developed that showed anti-inflammatory effects in a rat model of antigen-induced arthritis and could reduce inflammatory pain in vivo [69,70] (Table 1). It remains to be investigated if these substances could potentially be used as an anti-inflammatory treatment in patients, or if the pleiotropic effects of GPR4, especially in tumor biology, are confounding factors for the clinical use of GPR4 inhibitors. It must be monitored carefully what unwanted effects GPR4-inhibiting substances are causing and in which pathophysiological state their use is appropriate. If this can be controlled, there is a chance for GPR4-modulating substances that may be developed for clinical use.

## 3. TDAG8 (GPR65)

TDAG8 received its name and abbreviation, T-cell death-associated gene 8, as it was initially discovered as a novel coding gene in T cells [7]. Unlike the other proton-sensing GPCRs, TDAG8 is expressed almost exclusively in lymphoid tissues [71].

TDAG8 was identified as a receptor for the glycosphingolipid psychosine (*d*-galactosyl-*β*-1,1′-sphingosine). This suggested a role for TDAG8 in globoid cell formation as part of the pathophysiology of globoid cell leukodystrophy (GLD), which is characterized by apoptosis of oligodendrocytes and that involves psychosine signaling [72]. Its ability to act as a proton-sensing GPCR was discovered later. In 2005, Ishii et al. observed that TDAG8 can be activated by protons, at a pH lower than 7.2, causing a cAMP increase and ras homolog-gene-family-member A (RhoA) activation [42]. A monotonous increase in cAMP formation was reported over the whole investigated pH range of pH 7.2–5.8. However, this was an endpoint read after 30 min, which does not allow judgment on whether there is a time- or pH-dependent desensitization.

Since its discovery, several biological roles for TDAG8 have been implicated. For example, it was shown that acidic pH stimulation of TDAG8 causes reduction of IL-6 and tumor necrosis factor-α (TNFα) production after lipopolysaccharide (LPS) stimulation in peritoneal macrophages via a cAMP-dependent mechanism [73]. Likewise, TDAG8 activation reduces IL-6 and TNFα production in T cells and leads to elevated IL-10 production [74] and TDAG8-deficiency increases infiltration of neutrophils and macrophages during colonic inflammation [75]. These observations imply a rather anti-inflammatory and benign role of TDAG8 activation upon acidic pH stimulation in the context of inflammation.

In the context of pain, TDAG 8 was also found to be expressed in DRG-neurons [55], and its expression is strongly increased during carrageenan-induced transient and complete Freund’s adjuvant- (CFA-) induced chronic inflammatory pain 24 h after treatment [56]. Moreover, knockdown of TDAG8 reduced hyperalgesic priming and delayed the onset of inflammatory pain in mice [41], and TDAG8 may be involved in macrophage polarization during rheumatoid arthritis progression and related pain [76,77]. Likewise, TDAG8 shows increased expression in the spinal cord during bone-cancer-induced pain in vivo and increased cancer-induced pain in a PKA-dependent manner [78]. These results suggest an important but also ambiguous role of TDAG8 in inflammatory and cancer-induced pain.

In a similar context, TDAG8 was found to contribute to acidic citrate-induced itch together with TRPV1, and TDAG8-deficient mice had a markedly reduced response [79]. For all proton-sensing GPCRs, a convergence of signaling towards TRPV1 is important as this might facilitate its principal role for acid-induced pain in humans [25].

Concerning tumor biology, several contrasting roles for TDAG8 have been reported. For example, TDAG8 overexpression in Lewis lung carcinoma (LLC) was found to enhance tumor growth via PKA and extracellular signal-regulated kinase (ERK) [80]. Moreover, similar to GPR4, TDAG8 is overexpressed in human tumors, especially in colon, ovarian, and kidney tumor tissue [57]. On the other hand, TDAG8 activation by acidosis decreases the expression of the strong oncogene c-Myc in human lymphoma cells [81]. Based on the strong expression of TDAG8 in lymphoid tissues, it is considered to function as a contextual tumor suppressor and its activation may represent a potential anti-tumorigenic approach, specifically in hematological malignancies [82].

Apart from its involvement in tumor biology, other roles for TDAG8 have been described. TDAG8 has been shown to increase eosinophil viability in acidic pH via cAMP which is a hallmark mechanism for prolonging and aggravating inflammation in asthma [83]. Additionally, TDAG8 may protect from a brain injury after ischemia [84] and may as well be relevant for increasing bone density. It was observed that TDAG8 activation inhibits calcium resorption in osteoclasts and may thus enhance bone density [85] (Figure 2).

There are only a few substances known that may modulate the activity of TDAG8. Apart from protons and psychosine, an allosteric modulator, BTB09089, has been described as an activator for TDAG8 that displayed neuroprotective effects in a rat model of ischemic stroke [86]. Moreover, docking studies with multiple compounds have identified the compounds ZINC13684400 as a positive and ZINC62678696 as a negative allosteric modulator of TDAG8 [11] (Table 2). In conclusion, TDAG8 activation may have rather beneficial consequences in the context of inflammation, although this depends on the affected tissue and inflammatory microenvironment. However, TDAG8 activation may also increase the activity of sensory neurons and may increase inflammatory pain and cancer-induced pain. Specifically, there is still a lack of selective compounds for TDAG8, that may be used to decipher the biological roles of this receptor in more detail and to identify therapeutic approaches in TDAG8 modulation and signaling.

## 4. OGR1 (GPR68)

The OGR1 receptor was originally identified in a human ovarian cancer cell line that maps to a region of chromosome 14 and was therefore named ovarian cancer G-protein coupled receptor [87]. It was originally described as a receptor for sphingosylphosphorylcholine (SPC) but this publication was retracted [88].

Its role as a proton-sensing GPCR was first described by Ludwig et al., and the authors observed that the maximum activation of OGR1 by protons occurs at pH 6.8, mainly causing the production of inositol trisphosphate (IP_3_) [1]. They assessed the production of IP_3_ upon continuous stimulation every 10 min, which indicated a limited degree of desensitization. Like GPR4, OGR1 is widely expressed [89], for example in peripheral sensory neurons in dorsal root ganglia (DRG) [55], but its expression is unchanged in DRGs during transient or chronic inflammatory pain [56].

OGR1 has been suggested to be a mechanoreceptor activated by both cell stretch and acidosis [36]. A recent study observed the expression of OGR1 expressed in endothelial cells of small-diameter arteries and identified OGR1 as a mechanosensitive flow sensor that acts via G_q/11_-IP_3_-calcium and is a crucial signaling component in cardiovascular pathophysiology [37]. In a rodent model of myocardial infarction, OGR1 was found to be expressed in cardiomyocytes. These OGR1-positive cardiomyocytes formed a proton-sensing cellular zone surrounding the myocardial infarction. Moreover, 3,5-disubstituted isoxazoles (lsx), small molecules targeting Notch-activated epicardium-derived cells, activate the OGR1 receptor [90] which indicates a role for OGR1 during ischemic heart disease.

OGR1 is also expressed in human aortic smooth muscle cells (AoSMC) and can induce cAMP and cyclooxygenase-2 (COX2)-dependent prostaglandin I_2_ (PGI_2_) production upon extracellular pH reduction, which can cause vasodilatation [91]. Moreover, OGR1 seems to contribute to the development of asthma. It was observed that OGR1 expressed in human airway smooth muscle cells (ASMC) seems to mediate the synthesis of the proinflammatory cytokines interleukin 6 (IL-6), IL-8 and increase of intracellular calcium concentrations upon acidic pH stimulation, which may subsequently lead to enhanced formation of extracellular matrix proteins and increase the expression of connective tissue growth factor (CTGF) [9,92].

Likewise, OGR1-deficient mice show markedly reduced response in an ovalbumin-induced model of asthma. The authors of this study suggest that OGR1 specifically in dendritic cells is required for the full inflammatory response in this model [93]. Apart from its cardiovascular and respiratory properties, OGR1 was reported to have contrasting roles in tumor biology and has both tumor-suppressing and tumor-promoting functions. For example, overexpression of OGR1 in prostate cancer cells caused a reduction of metastasis to the lungs and spleen in vivo [94], and overexpression of OGR1 in ovarian cancer cells caused a reduction of proliferation and migration, and the cells showed stronger adhesion to the extracellular matrix proteins [95]. However, OGR1 is also widely expressed in human tumors and tumor cell lines [59,60] as well as in cancer-associated fibroblasts (CAFs) [96] and has been attributed to procarcinogenic effects by mediating the interaction between tumor cells and CAFs [97,98,99]. It may also be involved in the immunosuppression of prostate cancer cells. In this context, OGR1 may maintain tumor-associated macrophages in an M2-like state and inhibits T-cell infiltration, which promotes tumor growth [100], yet the exact mechanism for this observation is unclear.

Additional observations suggest an involvement of OGR1 in osteoclastogenesis. OGR1 may be a central acid sensor in bones, possibly by activation of the G_q_-PLC-pathway and intracellular increase of calcium concentrations [1,10,101]. Finally, OGR1 has also been described to be involved in insulin secretion under acidic conditions via G_q/11_ activation [102] (Figure 2).

Currently, there are only a few pharmacological OGR1-addressing substances known. Among the known agonists are several peptides that were identified in pairing studies [103], as well as the aforementioned 3,5-disubstituted isoxazoles [90]. Another study identified various positive allosteric modulators of OGR1. The most interesting compounds were ogerin, and the widely used benzodiazepine lorazepam [11], and MS48107, an ogerin-derived compound [104] (Table 3).

Similar to GPR4, OGR1 has several contrasting effects, in tumor biology, whereas its role in cardiovascular physiology and its role as a mechanoreceptor seems to be more prominent and distinct compared with the other proton-sensing GPCRs. However, these properties of OGR1 in the various disease states and tissues need to be understood in more detail to clearly decipher the role of OGR1 and to identify specific pathophysiological mechanisms that are mediated by this receptor, and that may lead to the development of OGR1 modulating substances for clinical use.

## 5. G2A (GPR132)

The abbreviation, G2A, is derived from an early observation, that G2A is a stress-induced receptor and that its expression is enhanced upon DNA damage, leading to cell cycle arrest in the G2/M phase. The term: “G2-cell cycle arrest” was chosen as a name for the receptor, for which at that time no other function was known [106]. G2A is mainly expressed in leukocytes and seems to be responsible for the migration of macrophages [8,107]. Interestingly, G2A-deficient mice develop a late-onset autoimmune disorder at an age above one year, that is characterized by aberrant lymphocytic infiltration into numerous tissues [108]. However, G2A is also expressed in peripheral sensory neurons that co-express the TRPV1 channel [55]. In these neurons, G2A seems to communicate with TRPV1 and enhance its activity via protein kinase C during oxaliplatin-induced neuropathic pain [109].

G2A shows the weakest proton sensitivity of the four receptors, its activation generates inositol trisphosphate (IP_3_) via G_q_-activation [5]. It is activated at pH 7.4, with a response monotonously increasing throughout the investigated pH range pH 8.2–6.6. There are no results that allow judging potential desensitization. A pH-sensing role in immune cells was questioned and rather assigned to TDAG8. G2A was suggested to be a receptor for lipids, particularly for lysophospholipids [6]. However, as with GPR4, the paper claiming G2A activation by LPC was later retracted [46]. It seems that lysophospholipids are not direct activators but are rather involved in G2A-trafficking to the plasma membrane [110]. Moreover, lysophosphoplipids seem to serve as chemoattractants for T-cell migration via G2A [111,112]. Another study concludes, that lysophsophatidylcholine (LPC) seems to be a weak antagonist of G2A, but the required concentrations are quite high (>10 µM) [5]. In 2005, Obinata and colleagues published a study that identified G2A as a receptor for oxidized lipids derived from linoleic acid (hydroxyoctadecadienoic acids, HODEs), or from arachidonic acid (hydroxyeicosatrienoic acids, HETEs). The strongest G2A activation was caused by the linoleic acid metabolite 9S-HODE (EC_50_: ~0.5 µM) in the heterologous expression system [39]. A recent study also identified N-palmitoylglycine and N-linoleoylglycine as G2A-activators with similar potency to 9-HODE, which strengthens the concept of G2A being a receptor for signaling lipids rather than protons and acidification. In the same study, the angiotensin-II type I receptor antagonist telmisartan as well as a telmisartan analogue, GSK1820795A, were identified as potent G2A inhibitors [113].

In the tumor microenvironment, a role for G2A has been suggested in the silencing of tumor-associated macrophages (TAMs). Breast tumor cells produce lactate which can activate G2A in TAMs causing intracellular activation of the peroxisome proliferator-activated receptor γ (PPARγ), which keeps macrophages in a non-aggressive M2-like state and enhances tumor growth [114,115]. In this regard, G2A may be a novel target for breast cancer, and G2A-inhibitors may interrupt the lactate-G2A-PPARγ-axis and may shift TAMs towards a proinflammatory phenotype in which they can attack tumor cells. In a similar context, G2A has previously been shown to enhance the oncogenic transformation of fibroblasts and enhances proliferation and actin rearrangement via G_α13_ and RhoA in 3T3 fibroblasts [116]. Likewise, G2A is widely expressed in tumor cell lines and human tumors [59,68].

Another important role of G2A has been suggested in hematopoiesis. The oxidized lipids 11,12-EET (epoxyeicoatrienoic acid) and 9,10-EpOME (epoxyoctadecadienoic acid) can activate G2A at high concentrations in heterologous expression systems, using recruitment of β-arrestin as readout. G2A seems to be required for normal marrow transplantation and G2A-deficient mice have a reduced number of hematopoietic stem cells compared with wild-type mice [40].

Moreover, a second-generation imipridone, ONC212, seems to activate G2A in the nanomolar range and initiates G_aq_ signaling. This compound was shown to induce cellular stress and apoptosis in acute myeloid leukemia (AML) cells and could reduce AML growth in vivo [117] (Table 4), suggesting that G2A may be a promising target for the treatment of acute myeloid leukemia.

In conclusion, G2A seems to differ profoundly from the other proton-sensing GPRCs in many respects: first, it has the weakest sequence similarity and shows the weakest activation by protons. Second, it is activated by oxidized fatty acid derivatives. Third, it has distinct roles in immune cell migration, hematopoiesis, and neuropathic pain that are unique in the group of proton-sensing GPCRs and make it an interesting therapeutic target in related disease states (Figure 2).

## 6. Outlook

### 6.1. Modulating and Targeting the Four GPCRs

All four proton-sensing GPCRs show an interesting overlapping property, apart from the proton-sensing capabilities: the modulation by lysophospholipids and sphingolipids (Figure 2). Although most of these interactions are probably allosteric or indirect, it is interesting that all four receptors share this property. This points out the significance of lipid-GPCR interactions and raises the question of whether the four proton-sensing GPCRs are also modulated by phospholipids or lipid rafts directly in the membrane, thus affecting their activity. Similar interactions are known for other membrane proteins, such as, for example, the interaction between potassium or transient receptor potential ion channels and the phosphatidylinositol phosphates [119,120,121].

It should also be kept in mind that the four receptors have been described to heterodimerize, thereby changing their properties [89]. For example, heterodimers of OGR1 and G2A can increase proton-induced intracellular calcium concentrations, compared with activating each receptor individually [122].

It is still puzzling why there is a redundancy of proton-sensing GPCRs with overlapping pH ranges in many tissues. One explanation could be that there are different steps of cellular responses and that a full cellular response requires activation of several GPCRs at the same time. In this regard, the heterodimerization may play a role and may change the pH range for receptor activation and alter its downstream signaling [89].

**Figure 3:**
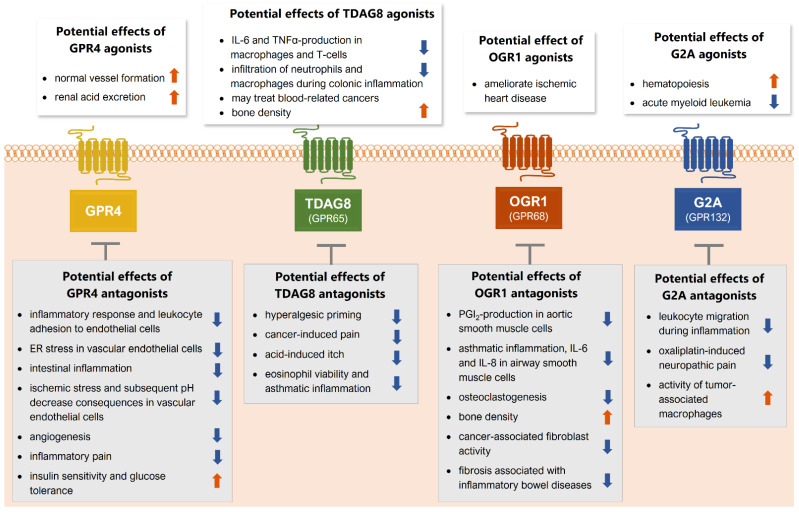
Potential therapeutic effects of agonists and antagonists of the four proton-sensing GPCRs. GPR4 (yellow), TDAG8 (GPR65, green), G2A (GPR132, blue), and OGR1 (GPR68, red). Abbreviations: ER: endoplasmic reticulum, IL-6: interleukin 6, TNFα: tumor necrosis factor α, PGI_2_: prostaglandin I_2_, IL-8: interleukin 8.

So far, the four receptors have shown a remarkable variety of biological properties and have been identified as potential targets for several pathophysiological states and diseases. For all four receptors, both agonists and antagonists could potentially reduce or ameliorate pathological states in multiple disease states (Figure 3), making it difficult to target them without causing side effects. These contrasting effects can be observed for all four receptors. It is therefore necessary that pharmacological strategies targeting one of the receptors need to be context-dependent and possible off-target effects need to be monitored carefully.

Unfortunately, only a few compounds are available that target GPR4, TDAG8, OGR1, and G2A, either as allosteric modulators or agonists and antagonist and for many of them the selectivity and specificity toward the respective receptor has not been shown sufficiently (Table 1, Table 2, Table 3 and Table 4). Therefore, the identification and characterization of novel pharmacological tools should be subject to future studies to fully understand the biology of the four receptors.

Until now, several genetic variants of the proton-sensing GPCRs with potential clinical consequences have been described. For example, a genetic variant of TDAG8 (GPR65) that leads to amino acid exchange and reduces activity and signaling of the receptor has been associated with alterations in lysosomal pH and lysosomal dysfunction in the pathophysiological context of inflammatory bowel disease [123]. Likewise, another single nucleotide polymorphism in the TDAG8 gene has been associated with intestinal inflammation in patients [124]. However, the exact mechanistic and cellular consequences of this mutation are unknown.

For OGR1 (GPR68), several rare homozygous variants were described to be associated with loss of function of the receptor and reduced dental enamel formation in humans [125].

For the human G2A (GPR132), two alternative splice variants were reported. One of them shows higher basal activity leading to increased intracellular IP formation but there is no difference in the response to the ligand 9-HODE compared with the original variant [126]. The physiological effects of this splice variant are yet to be determined.

The effects of the variants for TDAG8 and OGR1 that are associated with reduced activity and signaling of the receptors, point towards possible side effects of antagonists and should be considered carefully upon clinical development and testing of such substances.

### 6.2. Expanding the Group of Proton-Sensing GPCRs

When comparing the biological properties of the four receptors, their pleiotropic effects in many different pathophysiological states and tissues become apparent. This raises the question of whether the umbrella term “proton-sensing GPCRs” encloses sufficiently similar members to functionally consider this as a family. It may thus be more suitable to regard the four receptors individually, with proton-sensing being an overlapping property shared by all four receptors. Indeed, there are other distinct functions of the receptors that can be observed. For example, OGR1 seems to be a mechanoreceptor in the vascular system [36,37] and G2A seems to be a receptor for oxidized fatty acids rather than protons [39,40]. Moreover, other receptors may as well be activated by protons. In a recent publication, the orphan receptors GPR31 and GPR151 were found to be activated under acidic conditions in vitro [127]. Although these two receptors have only recently been identified as potential proton-sensing GPCRs, their physiological roles have partly been described previously. Interestingly, GPR31 has been identified as a receptor for the eicosanoid 12-HETE (hydroxyeicosatetraenoic acid) [128] and thus shares the property of being a lipid receptor with the other GPCRs.

In the intestinal lumen, bacteria secrete pyruvate and lactate causing activation of GPR31 in CX3CR1+ immune cells leading to their dendrite protrusion and enhancing intestinal immune responses [129]. Moreover, the 12-HETE-GPR31 interaction has recently been described as a crucial process in the development of liver ischemia–reperfusion (IR) injury [130], and the 12(S)-HETE dependent GPR31 activation was shown to stimulate thrombin-PAR4 platelet activation and arterial thrombosis in human platelets via Gi activation, suggesting inhibition of this system as a beneficial strategy for the prevention of arterial thrombosis [131]. In the context of tumor biology, GPR31 was identified to mediate KRAS membrane association and is crucial for proliferation and survival of KRAS-dependent tumors suggesting that GPR31 may represent a target for anti-RAS therapy [132]. In contrast, GPR151 seems to be expressed mainly in. In contrast, GPR151 seems to be expressed mainly in the nervous system and plays a role in the development of neuropathic pain (see next section).

These findings indicate that proton-sensing is only one of many properties of the GPCRs discussed here and that other proton-sensing GPCRs may exist that allow detection of a broader pH range. A similar concept has been described for the acid-sensing ion channels (ASICs) that are also activated by acidification and have distinct but also overlapping pH ranges for their activation [133,134].

While the four receptors were initially thought to contribute mainly to inflammatory processes due to their proton-sensing properties, the observations described above suggest a much broader role of these proteins in different aspects of health and disease which needs to be addressed by future studies.

### 6.3. Proton-Sensing GPCRs in Persistent Pain States

As indicated above, GPR4, TDAG8, and G2A share a role in the pathophysiology of persistent pain states. GPR4 was found to be involved in chronic inflammatory pain and GPR4-inhibitors could reduce arthritis-induced inflammatory pain in a rodent model [69,70]. Likewise, TDAG8 was shown to play a role in chronic inflammatory pain, as it seems to modulate macrophage polarization in the context of arthritis-induced inflammatory pain [76,77]. It also seems to be involved in hyperalgesic priming at the onset of inflammatory pain [41]. Moreover, TDAG8 seems to contribute to cancer-induced pain via PKA activation in spinal microglia [78]. In contrast, G2A seems to contribute to oxaliplatin-induced mechanical hypersensitivity via interaction with the TRPV1 channel in sensory neurons and mediates macrophage migration by activation of the Toll-like receptor 4 (TLR4) pathway in early nerve-injury-induced neuropathic pain [109,135].

GPR151, which has recently been identified as novel proton-sensing GPCR, is mainly expressed in the nervous system. It seems to contribute to neuropathic pain in particular. A recent study proposed a role for GPR151 in the maintenance of trigeminal neuropathic pain via Gαi-coupling, induction of ERK, and subsequent neuroinflammation [136]. Mechanistically, GPR151 seems to interact with the purinergic receptor P2X3 in sensory neurons. As a consequence, the activity of P2X3 increases causing enhanced activity of sensory neurons and subsequent activation of spinal microglia under neuropathic pain conditions [137].

In summary, preclinical data point towards a critical role of GPR4, TDAG8, G2A, and GPR151 in the onset, and maintenance of persistent pathophysiological pain and antagonists of these receptors may be promising novel therapeutics for the treatment of inflammatory and neuropathic pain. More specifically, GPR4 seems to be a target candidate for inflammatory pain, TDAG8 for inflammatory and cancer-induced pain, and G2A and GPR151 seem to be particularly involved in the development of neuropathic pain. Moreover, since most of these four receptors can be activated by lipid mediators, targeting synthesis or release of these lipids may represent an alternative strategy for the treatment of pain.

## Figures and Tables

**Table 1:** Agonists and antagonists of GPR4 known so far. Abbreviations: EC_50_/IC_50_: half-maximal excitatory or inhibitory concentration, cAMP: cyclic adenosine monophosphate, HEK: human embryonic kidney, CFA: complete Freund’s adjuvant, refs: references.

**Agonist**	**EC_50_ or Active Concentration**	**Test System and/or Consequence**	**Refs**
Protons	pH 7.6–5.6	pH-dependent cAMP assay in various transfected cell lines	[1,62,63]
**Antagonist**	**IC_50_ or Active Concentration**	**Test System and Consequence**	**Refs**
Compound 3b (dibenzazepine derivative)	IC_50_: 67 nM	HEK-293-cell-based Luciferase assay, mouse myocardial infarction model	[66]
NE 52-QQ57 (compound 13 in reference)	IC_50_: 70 nM	pH-dependent cAMP assay in HeLa and HEK-293 cells, angiogenesis growth factor model, CFA-model for inflammatory pain	[69]
Compound 39c (imidazopyridine derivative)	IC_50_: 110 nM	pH-dependent cAMP assay in HeLa and HEK-293 cells, mouse VEGF-angiogenesis model, rat antigen-induced arthritis model, CFA-induced inflammatory pain	[70]

**Table 2:** Agonists, positive allosteric modulators (PAMs), and negative allosteric modulators of TDAG8 (GPR65) known so far. Abbreviations: EC_50_ / IC_50_: half-maximal excitatory or inhibitory concentration, cAMP: cyclic adenosine monophosphate, CHO: Chinese hamster ovary, HEK: Human embryonic kidney, refs: references.

**Agonist**	**EC_50_ or Active Concentration**	**Test System and/or Consequence**	**Refs**
Protons	pH 7.2–5.7	pH-dependent cAMP assay in stably TDAG8-transfected CHO cells	[42]
Psychosine	EC_50_: 3.4 µM (cAMP assay)	cAMP assay, calcium-mobilization in TDAG8-transfected HEK-293 cells	[72]
BTB09089	active concentration > 5 µM	cAMP assay in splenocytes	[74]
**Positive Allosteric Modulators (PAMs)**	**IC_50_ or Active Concentration**	**Test System and Consequence**	**Refs**
ZINC13684400	micromolar range	library of drugs and compounds tested in a yeast TDAG8 expressing system, using cAMP production as readout	[11]
**Negative Allosteric Modulators (NAMs)**	**IC_50_ or Active Concentration**	**Test System and Consequence**	**Refs**
ZINC62678696	micromolar range	library of drugs and compounds tested in a yeast TDAG8 expressing system, using cAMP production as readout	[11]

**Table 3:** Agonists and positive allosteric modulators (PAMs) of OGR1 (GPR68) known so far. Abbreviations: EC_50_/IC_50_: half-maximal excitatory or inhibitory concentration, cAMP: cyclic adenosine monophosphate, HEK: Human embryonic kidney, refs: references.

**Agonist**	**EC_50_ or Active Concentration**	**Test System and/or Consequence**	**Refs**
Protons	pH 7.8–5.6, maximum activity at pH 6.8	pH-dependent cAMP assay in various cells	[1,105]
3,5-disubstituted isoxazoles	micromolar range	Calcium transients in transfected Notch-activated epicardium-derived cells (NECs)	[90]
CART(42–89)(9–28) shorter variant of cocaine- and amphetamine-regulated transcript	EC_50_: 1 µM	Identified via: dynamic mass redistribution assay pathway-independent receptor internalization assay β-arrestin recruitment assay	[103]
steocrin-derived peptide (115–133)	EC_50_: 380 nM		[103]
pro-opiomelanocortin-derived peptide (141–162)	EC_50_: 1.3 µM		[103]
**Positive Allosteric Modulators (PAMs)**	**IC_50_ or Active Concentration**	**Test System and Consequence**	**Refs**
ogerin	K_b_: ~10 µM, requires the presence of protons	library of drugs and compounds tested in a yeast OGR1 expressing system, using cAMP production as readout	[11]
lorazepam	non-selective, micromolar range		[48]
MS48107	K_b_: ~1–10 µM	cAMP assay in transfected HEK-293 cells.	[104]

**Table 4:** Strongest agonists and antagonists of G2A (GPR132).

**Agonist**	**EC_50_ or Active Concentration**	**Test System and/or Consequence**	**Refs**
Protons	pH 8.2–6.6	Gq-activation, generation of IP3 in NIH-3T3 fibroblasts	[5]
9S-HODE	EC_50_: ~0.5 µM [118]	[Ca2+]i-increase in stably G2A-transfected CHO cells	[39,118]
11-HETE	EC_50_: ~1 µM [118]	[Ca2+]i-increase in stably G2A-transfected CHO cells	[39,118]
N-palmitoylglycine	EC_50_: ~800 nM, similar for human- rat- and mouse-G2A	yeast assay and β-arrestin association assay (in HEK-293 cells)	[113]
N-linoleoylglycine	EC_50_: ~800 nM, similar for human- rat- and mouse-G2A	yeast assay and β-arrestin association assay (in HEK-293 cells)	[113]
ONC212 (second-generation imipridone)	~400 nM	PathHunter β-arrestin association assay in HEK-293 cells	[117]
11,12-EET	~10 µM	PathHunter β-arrestin association assay in HEK-293 cells	[40]
9,10-EpOME	~10 µM	PathHunter β-arrestin association assay in HEK-293 cells	[40]
**Antagonist**	**IC_50_ or Active Concentration**	**Test System and Consequence**	**Refs**
Lysophosphatidylcholine (LPC)	~10 µM	inhibits Gq-dependent, generation of IP3 in NIH-3T3 fibroblasts	[5]
Telmisartan	~10 µM	β-arrestin association assay in HEK-293 cells	[113]
GSK1820795A	~1 µM	β-arrestin association assay in HEK-293 cells	[113]

This table includes the strongest G2A agonists and antagonists with EC_50_ and IC_50_ concentrations up to 10 µM. Abbreviations: EC_50_/IC_50_: half-maximal excitatory or inhibitory concentration, HODE: hydroxyoctadecadienoic acid, HETE: hydroxyeicosatrienoic acid, EpOME: epoxyoctadecadienoic acid, EET: epoxyeicoatrienoic acid, CHO: Chinese hamster ovary cells, HEK: human embryonic kidney, IP_3_: inositol trisphosphate, refs: references.
